# Prime editing optimized RTT permits the correction of the c.8713C>T mutation in *DMD* gene

**DOI:** 10.1016/j.omtn.2022.09.022

**Published:** 2022-10-02

**Authors:** Cedric Happi Mbakam, Joel Rousseau, Yaoyao Lu, Anne Bigot, Kamel Mamchaoui, Vincent Mouly, Jacques P. Tremblay

**Affiliations:** 1 CHU de Québec Research Centre, Laval University, Québec, QC G1V 0A6, Canada; 2 Molecular Medicine Department, Faculty of Medicine, Laval University, Québec, QC G1V 4G2, Canada; 3 Myology Research Center, Institute of Myology, 75013 Paris, France

**Keywords:** MT: RNA/DNA editing, prime editing, CRISPR-Cas9, RTT, Duchenne muscular dystrophy, *DMD* gene, c.8713C>T mutation

## Abstract

Duchenne muscular dystrophy is a severe debilitating genetic disease caused by different mutations in the *DMD* gene leading to the absence of dystrophin protein under the sarcolemma. We used CRISPR-Cas9 prime editing technology for correction of the c.8713C>T mutation in the *DMD* gene and tested different variations of reverse transcription template (RTT) sequences. We increased by 3.8-fold the editing percentage of the target nucleotide located at +13. A modification of the protospacer adjacent motif sequence (located at +6) and a silent mutation (located at +9) were also simultaneously added to the target sequence modification. We observed significant differences in editing efficiency in interconversion of different nucleotides and the distance between the target, the nicking site, and the additional mutations. We achieved 22% modifications in myoblasts of a DMD patient, which led to dystrophin expression detected by western blot in the myotubes that they formed. RTT optimization permitted us to improve the prime editing of a point mutation located at +13 nucleotides from the nick site to restore dystrophin protein.

## Graphical abstract

**Figure:**
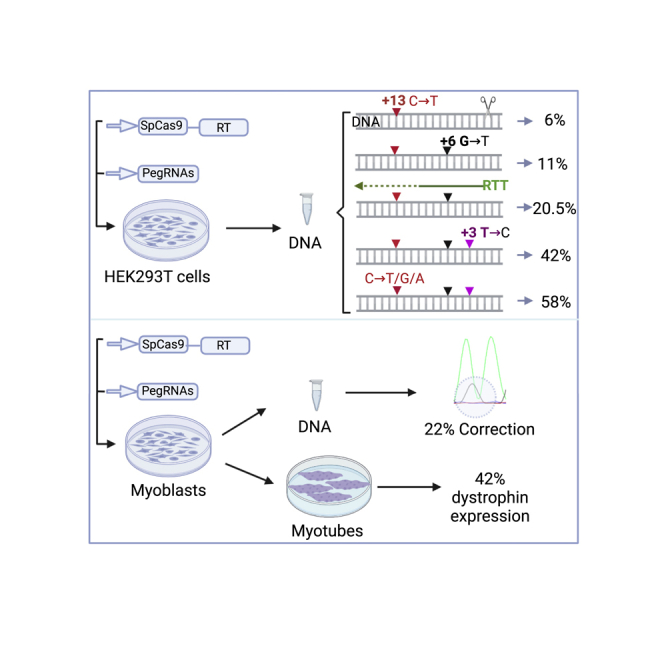

## Teaser

Duchenne muscular dystrophy is a severe muscle-wasting disease caused by mutations in the *DMD* gene. Optimized prime editing permitted us to achieve 22% correction of c.8713C>T mutation in *DMD* exon 59 in a patient myoblast. This gene modification led to 42% dystrophin expression detected by western blotting of myotubes.

## Introduction

Duchenne muscular dystrophy (DMD) is a lethal X-linked disease characterized by progressive muscle wasting with high burden on patients and family members.^1^, ^2^, ^3^, ^4^ The prevalence is estimated to 19.8 per 100,000 live male births, 7.1 cases per 100,000 males, and 2.8 cases per 100,000 individuals in the general population.^5^ The prevalence is also evaluated at 10.9, 6.1, 2.2, and 1.9 per 100,000 males respectively in France, Canada, the United Kingdom, and the United states.^4^ The disease is caused by mutations in the *DMD* gene coding for the dystrophin protein, which is normally located under the sarcolemma.^6^^,^^7^ Different types of mutations leading to DMD have been identified in the *DMD* gene, which is one of the biggest human genes,^8^ and different therapeutic strategies have been developed.^9^^,^^10^ These mutations include exonic and intronic duplications accounting for 10%–15% of DMD mutations, small insertions and deletions (3%), point mutations (nonsense and missense mutations, splice site mutations, and mid intronic mutations, 26%) and single- or multi-exon deletions (60%–70%).^7^^,^^11^ Many research groups have been using CRISPR-Cas9 genome editing to modify the *DMD* gene to restore the dystrophin expression.^12^, ^13^, ^14^, ^15^, ^16^, ^17^, ^18^, ^19^ The CRISPR-Cas9 technology uses a specific single guide RNA (sgRNA) to target and cut DNA at a desired site to induce different types of modifications following DNA repair by non-homologous end-joining or homology-directed repair (HDR).^20^ HDR-mediated DMD correction has been shown in a canine model of DMD to be associated with a set of challenges affecting the editing efficiency.^21^ The recent CRISPR-Cas9 prime editing technique is more precise and permits base-to-base conversion, replacement, and insertion and deletion in the genome.^22^, ^23^, ^24^, ^25^, ^26^ For prime editing, the SpCas9 (*Streptococcus pyogenes*) nuclease has been modified into an SpCas9 nickase (SpCas9n) and is fused with an engineered reverse transcriptase from *Murine leukemia virus*. Prime editing also required a modified sgRNA called prime editing guide RNA (pegRNA).^22^ Prime editing has already been used to correct *DMD* gene mutations located close to the SpCas9n nick site.^14^^,^^19^^,^^27^ However, the efficacy of prime editing decays rapidly when the target nucleotide is far from the nick site. Our study aimed to improve the prime editing effectiveness for the correction of c.8713C>T point mutation in exon 59 of the *DMD* gene, which is positioned far from the nick site, i.e., at +13 from the nick site.

## Results

### Verifying whether prime editing permits a specific modification at +13 from the nick site

**Figure 1:**
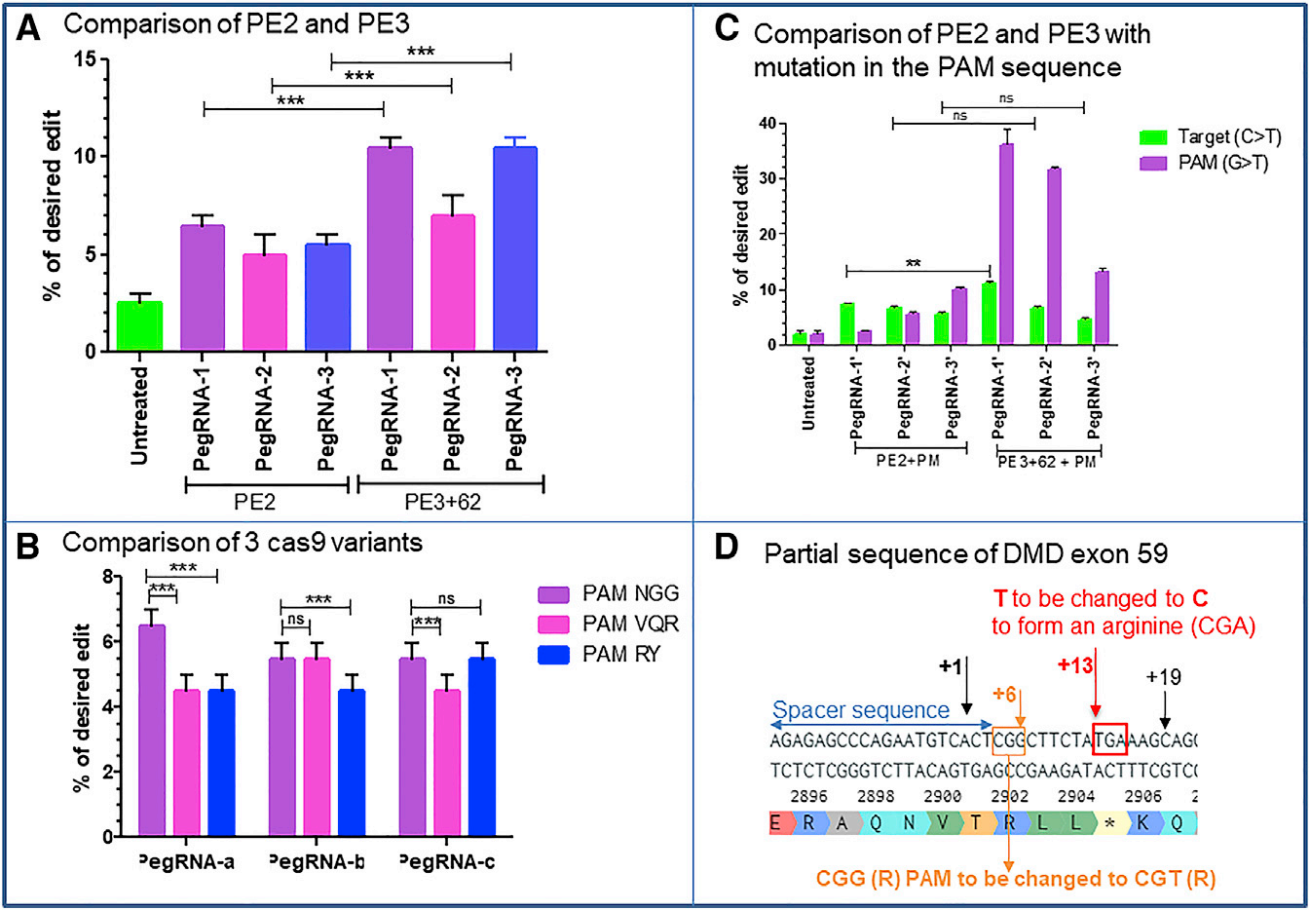
PE2 and PE3 editing of *DMD* exon 59 using SpCas9 and variants (A) Editing efficiency using the initial three pegRNAs for the PE2 and PE3 strategies (using an sgRNA inducing a nick at position +62) to induce c.8713C>T mutation in exon 59 of *DMD* gene. The differences between pegRNA1, pegRNA2, and pegRNA3 for PE2 and PE3 were statistically significant (∗∗∗p < 0.001). (B) Results obtained with three different pegRNAs (a, b, and c) designed individually for each nuclease variant recognizing the NGG PAM (for SpCas9), the NGAN PAM (for SpCas9-VQR), and the NNN PAM (for SpCas9-RY). ns indicates that the differences between the pegRNAs of these Cas9 variants were not significant. The asterisks indicate that the differences were statistically significant (∗∗∗p < 0.001). (C) Editing efficiency for PE2 and PE3 strategies using three pegRNAs containing two mutations each: the target mutation and the mutation in the PAM sequence (PM). The difference of mutation at the target site was significant only for pegRNA1 used for PE2 and PE3. (D) Partial sequence of *DMD* exon 59 carrying a nonsense mutation to be corrected (TGA sequence shown by the red square at the position +13). The red square is the TGA stop codon to be corrected to a CGA codon which is an arginine (R). The orange square contains the sequence of the PAM CGG to be modified to CGT to increase the editing efficiency of the nonsense mutation at the position +13. The numbers +1 and +13 represent different positions from the nick site, and the blue arrow is the 20-nt spacer sequence. These experiments were done in independent triplicates (n = 3). The non-parametric Mann-Whitney U test was performed to calculate the p values.

We initially verified whether prime editing could induce a nucleotide mutation in exon 59 of *DMD* gene to change a stop codon (TGA) at position 8,713 into an arginine codon (CGA) to restore the dystrophin protein expression. Since at the beginning of the project we did not have myoblasts containing that mutation, we initially decided to induce a c.8713C>T mutation to create a stop codon instead of inducing the correction of the mutation. The cytidine (C) nucleotide of CGA codon coding for an arginine amino acid (R) had to be changed into a thymine (T) nucleotide to form the TGA stop codon located at the position +13 from the closest SpCas9 possible nick site (Figure 1D). This is considered to be a little too far from the nick site and thus at a less efficient position for the nucleotide modification.^22^ We designed three pegRNA sequences (Table 1, rows 1A) named pegRNA1 (RTT16, PBS14), pegRNA2 (RTT15, PBS12), and pegRNA3 (RTT15, PBS16) for the rapid screening of targeted nucleotide modification. For the PE2-NGG strategy,^22^ the HEK293T cells were co-transfected with pCMV-PE2 plasmid (Addgene #132775) coding for the normal SpCas9n (using an NGG protospacer adjacent motif [PAM]) fused with the reverse transcriptase and pU6-pegRNA-GG-acceptor plasmid (Addgene #132777) coding for one of the pegRNA constructs. Three days after the transfection, a part of exon 59 of *DMD* gene was PCR amplified from harvested cells using a pair of primers (Table 2) and Sanger sequenced. The results indicated that the editing percentages were 6.5% ± 0.7%, 5% ± 1.4%, and 5.5% ± 0.7% for pegRNA1, pegRNA2, and pegRNA3, respectively (Figure 1A). For the PE3 strategy,^22^ we inserted an additional sgRNA to the pBSU6 plasmid to induce a second nick at position +62 from the initial nick site by the pegRNA. We co-transfected HEK293T cells with the pCMV-PE2 plasmid, the pU6-pegRNA-GG-acceptor plasmid, and the pBSU6 plasmid. The results showed 10.5% ± 0.7%, 7% ± 1.4%, and 10.5% ± 0.7% editing percentage, respectively for pegRNA1, pegRNA2, and pegRNA3 (Figure 1A).

**Table 1:** pegRNA sequences

Names	Spacer sequences	PBS sequences	RTT sequences	sgRNA for PE3
**Initial experiments in HEK293T (1A)**				

pegRNA1	GAGAGAGCCCAGAATGTCACT	GACATTCTGGGCTC	TTCATAGAAGCCGAGT	GTCTGCCAGTCAGCGGAGTGC
pegRNA2	GAGAGAGCCCAGAATGTCACT	GACATTCTGGGC	TC**A**TAGAAGCCGAGT	GTCTGCCAGTCAGCGGAGTGC
pegRNA3	GAGAGAGCCCAGAATGTCACT	GACATTCTGGGCTCTC	TC**A**TAGAAGCCGAGT	GTCTGCCAGTCAGCGGAGTGC

**Cas9 variants experiments in HEK293T (1B)**				

pegRNAa-VQR	GAGAATGTCACTCGGCTTCTA	AAGCCGAGTG	CCTGCTTTC**A**TAG	GTCTGCCAGTCAGCGGAGTGC
pegRNAb-VQR	GAGAATGTCACTCGGCTTCTA	AAGCCGAGTGACA	CCTGCTTTC**A**TAG	GTCTGCCAGTCAGCGGAGTGC
pegRNAc-VQR	GAGAATGTCACTCGGCTTCTA	AAGCCGAGTGACATTC	CCTGCTTTC**A**TAG	GTCTGCCAGTCAGCGGAGTGC
pegRNAa-RY	GATGTCACTCGGCTTCTACGA	TAGAAGCCGAGTGA	AGCCTGCTTTC**A**	GCCTAAAACCTTGTCATATTG
pegRNAb-RY	GATGTCACTCGGCTTCTACGA	TAGAAGCCGAGTGACA	TCAGCCTGCTTTC**A**	GCCTAAAACCTTGTCATATTG
pegRNAc-RY	GATGTCACTCGGCTTCTACGA	TAGAAGCCGAGTGAC	TCCTCAGCCTGCTTTC**A**	GCCTAAAACCTTGTCATATTG

**PAM modification experiments in HEK293T (1C)**				

pegRNA1′(16-14)	GAGAGAGCCCAGAATGTCACT	GACATTCTGGGCTC	TTC**A**TAGAAG**a**CGAGT	GTCTGCCAGTCAGCGGAGTGC
pegRNA2′(15-12)	GAGAGAGCCCAGAATGTCACT	GACATTCTGGGC	TC**A**TAGAAG**a**CGAGT	GTCTGCCAGTCAGCGGAGTGC
pegRNA3′(15-16)	GAGAGAGCCCAGAATGTCACT	GACATTCTGGGCTCTC	TC**A**TAGAAG**a**CGAGT	GTCTGCCAGTCAGCGGAGTGC

**Nucleotide position from the PAM experiments in HEK293T (1D)**				

+13C>T	GAGAGAGCCCAGAATGTCACT	GACATTCTGGGCTC	TTC**A**TAGAAG**a**CGAGT	GTCTGCCAGTCAGCGGAGTGC
+12A>T	GAGAGAGCCCAGAATGTCACT	GACATTCTGGGCTC	TTCG**A**AGAAG**a**CGAGT	GTCTGCCAGTCAGCGGAGTGC
+11T>A	GAGAGAGCCCAGAATGTCACT	GACATTCTGGGCTC	TTCGT**T**GAAG**a**CGAGT	GTCTGCCAGTCAGCGGAGTGC
+10C>T	GAGAGAGCCCAGAATGTCACT	GACATTCTGGGCTC	TTCGTA**A**AAG**a**CGAGT	GTCTGCCAGTCAGCGGAGTGC
+9T>A	GAGAGAGCCCAGAATGTCACT	GACATTCTGGGCTC	TTCGTAG**T**AG**a**CGAGT	GTCTGCCAGTCAGCGGAGTGC
+8T>A	GAGAGAGCCCAGAATGTCACT	GACATTCTGGGCTC	TTCGTAGA**T**G**a**CGAGT	GTCTGCCAGTCAGCGGAGTGC
+7C>T	GAGAGAGCCCAGAATGTCACT	GACATTCTGGGCTC	TTCGTAGAA**Aa**CGAGT	GTCTGCCAGTCAGCGGAGTGC
+5G>T	GAGAGAGCCCAGAATGTCACT	GACATTCTGGGCTC	TTCGTAGAAG**aA**GAGT	GTCTGCCAGTCAGCGGAGTGC
+4C>T	GAGAGAGCCCAGAATGTCACT	GACATTCTGGGCTC	TTCGTAGAAG**a**C**A**AGT	GTCTGCCAGTCAGCGGAGTGC
+3T>A	GAGAGAGCCCAGAATGTCACT	GACATTCTGGGCTC	TTCGTAGAAG**a**CG**T**GT	GTCTGCCAGTCAGCGGAGTGC
+2C>T	GAGAGAGCCCAGAATGTCACT	GACATTCTGGGCTC	TTCGTAGAAG**a**CGA**A**T	GTCTGCCAGTCAGCGGAGTGC
+1A>T	GAGAGAGCCCAGAATGTCACT	GACATTCTGGGCTC	TTCGTAGAAG**a**CGAG**A**	GTCTGCCAGTCAGCGGAGTGC

**Other nucleotides than PAM experiments in HEK293T (1E)**				

+1A>T	GAGAGAGCCCAGAATGTCACT	GACATTCTGGGCTC	TTC**A**TAGAAGCCGAG**a**	GTCTGCCAGTCAGCGGAGTGC
+2C>T	GAGAGAGCCCAGAATGTCACT	GACATTCTGGGCTC	TTC**A**TAGAAGCCGA**a**T	GTCTGCCAGTCAGCGGAGTGC
+3T>A	GAGAGAGCCCAGAATGTCACT	GACATTCTGGGCTC	TTC**A**TAGAAGCCG**t**GT	GTCTGCCAGTCAGCGGAGTGC
+4C>T	GAGAGAGCCCAGAATGTCACT	GACATTCTGGGCTC	TTC**A**TAGAAGCC**a**AGT	GTCTGCCAGTCAGCGGAGTGC
+5G>T	GAGAGAGCCCAGAATGTCACT	GACATTCTGGGCTC	TTC**A**TAGAAGC**a**GAGT	GTCTGCCAGTCAGCGGAGTGC
+6G>T	GAGAGAGCCCAGAATGTCACT	GACATTCTGGGCTC	TTC**A**TAGAAG**a**CGAGT	GTCTGCCAGTCAGCGGAGTGC
+7C>T	GAGAGAGCCCAGAATGTCACT	GACATTCTGGGCTC	TTC**A**TAGAA**a**CCGAGT	GTCTGCCAGTCAGCGGAGTGC
+8T>A	GAGAGAGCCCAGAATGTCACT	GACATTCTGGGCTC	TTC**A**TAGA**t**GCCGAGT	GTCTGCCAGTCAGCGGAGTGC
+9T>A	GAGAGAGCCCAGAATGTCACT	GACATTCTGGGCTC	TTC**A**TAG**t**AGCCGAGT	GTCTGCCAGTCAGCGGAGTGC
+10C>T	GAGAGAGCCCAGAATGTCACT	GACATTCTGGGCTC	TTC**A**TA**a**AAGCCGAGT	GTCTGCCAGTCAGCGGAGTGC
+11T>A	GAGAGAGCCCAGAATGTCACT	GACATTCTGGGCTC	TTC**A**T**t**GAAGCCGAGT	GTCTGCCAGTCAGCGGAGTGC
+12A>T	GAGAGAGCCCAGAATGTCACT	GACATTCTGGGCTC	TTC**Aa**AGAAGCCGAGT	GTCTGCCAGTCAGCGGAGTGC
+14G>T	GAGAGAGCCCAGAATGTCACT	GACATTCTGGGCTC	TT**aA**TAGAAGCCGAGT	GTCTGCCAGTCAGCGGAGTGC
+15A>T	GAGAGAGCCCAGAATGTCACT	GACATTCTGGGCTC	T**a**C**A**TAGAAGCCGAGT	GTCTGCCAGTCAGCGGAGTGC
+16A>T	GAGAGAGCCCAGAATGTCACT	GACATTCTGGGCTC	**A**TC**A**TAGAAGCCGAGT	GTCTGCCAGTCAGCGGAGTGC
+17A>T	GAGAGAGCCCAGAATGTCACT	GACATTCTGGGCTC	GC**a**TTC**A**TAGAAGCCGAGT	GTCTGCCAGTCAGCGGAGTGC
+18G>T	GAGAGAGCCCAGAATGTCACT	GACATTCTGGGCTC	G**a**TTTC**A**TAGAAGCCGAGT	GTCTGCCAGTCAGCGGAGTGC
+19C>T	GAGAGAGCCCAGAATGTCACT	GACATTCTGGGCTC	**a**CTTTC**A**TAGAAGCCGAGT	GTCTGCCAGTCAGCGGAGTGC

**Type of nucleotide in PAM experiments in HEK293T (1F)**				

+6G>A	GAGAGAGCCCAGAATGTCACT	GACATTCTGGGCTC	TTC**A**TAGAAG**t**CGAGT	GTCTGCCAGTCAGCGGAGTGC
+6G>C	GAGAGAGCCCAGAATGTCACT	GACATTCTGGGCTC	TTC**A**TAGAAG**g**CGAGT	GTCTGCCAGTCAGCGGAGTGC

**RTT length modification experiments in HEK293T (1G)**				

RTT13	GAGAGAGCCCAGAATGTCACT	GACATTCTGGGCTC	**A**TAGAAGCCGAGTGACATT CTGGGCTC	GTCTGCCAGTCAGCGGAGTGC
RTT15	GAGAGAGCCCAGAATGTCACT	GACATTCTGGGCTC	TCGTAGAAGCCGAGTGAC ATTCTGGGCTC	GTCTGCCAGTCAGCGGAGTGC
RTT16	GAGAGAGCCCAGAATGTCACT	GACATTCTGGGCTC	TTC**A**TAGAAGCCGAGTGAC ATTCTGGGCTC	GTCTGCCAGTCAGCGGAGTGC
RTT18	GAGAGAGCCCAGAATGTCACT	GACATTCTGGGCTC	CTTTCGTAGAAGCCGAGTG ACATTCTGGGCTC	GTCTGCCAGTCAGCGGAGTGC
RTT19	GAGAGAGCCCAGAATGTCACT	GACATTCTGGGCTC	GCTTTC**A**TAGAAGCCGAGT GACATTCTGGGCTC	GTCTGCCAGTCAGCGGAGTGC
RTT21	GAGAGAGCCCAGAATGTCACT	GACATTCTGGGCTC	CTGCTTTCGTAGAAGCCGA GTGACATTCTGGGCTC	GTCTGCCAGTCAGCGGAGTGC
RTT22	GAGAGAGCCCAGAATGTCACT	GACATTCTGGGCTC	CCTGCTTTC**A**TAGAAGCCG AGTGACATTCTGGGCTC	GTCTGCCAGTCAGCGGAGTGC
RTT24	GAGAGAGCCCAGAATGTCACT	GACATTCTGGGCTC	AGCCTGCTTTCGTAGAAGC CGAGTGACATTCTGGGCTC	GTCTGCCAGTCAGCGGAGTGC
RTT25	GAGAGAGCCCAGAATGTCACT	GACATTCTGGGCTC	CAGCCTGCTTTC**A**TAGAAG CCGAGTGACATTCTGGGCTC	GTCTGCCAGTCAGCGGAGTGC
RTT28	GAGAGAGCCCAGAATGTCACT	GACATTCTGGGCTC	CCTCAGCCTGCTTTC**A**TAG AAGCCGAGTGACATTCTG GGCTC	GTCTGCCAGTCAGCGGAGTGC
RTT31	GAGAGAGCCCAGAATGTCACT	GACATTCTGGGCTC	CCTCCTCAGCCTGCTTTC**A**T AGAAGCCGAGTGACATTC TGGGCTC	GTCTGCCAGTCAGCGGAGTGC
RTT35	GAGAGAGCCCAGAATGTCACT	GACATTCTGGGCTC	TTGACCTCCTCAGCCTGCT TTC**A**TAGAAGCCGAGTGA CATTCTGGGCTC	GTCTGCCAGTCAGCGGAGTGC
RTT39	GAGAGAGCCCAGAATGTCACT	GACATTCTGGGCTC	AGTATTGACCTCCTCAGCC TGCTTTC**A**TAGAAGCCGA GTGACATTCTGGGCTC	GTCTGCCAGTCAGCGGAGTGC
RTT42	GAGAGAGCCCAGAATGTCACT	GACATTCTGGGCTC	ACTCAGTATTGACCTCCTC AGCCTGCTTTC**A**TAGAAG CCGAGTGACATTCTGGGCTC	GTCTGCCAGTCAGCGGAGTGC

**RTT length and PAM modification experiments in HEK293T (1H)**				

RTT13	GAGAGAGCCCAGAATGTCACT	GACATTCTGGGCTC	**A**TAGAAG**a**CGAGTGACAT TCTGGGCTC	GTCTGCCAGTCAGCGGAGTGC
RTT15	GAGAGAGCCCAGAATGTCACT	GACATTCTGGGCTC	TCGTAGAAG**a**CGAGTGAC ATTCTGGGCTC	GTCTGCCAGTCAGCGGAGTGC
RTT16	GAGAGAGCCCAGAATGTCACT	GACATTCTGGGCTC	TTC**A**TAGAAG**a**CGAGTGA CATTCTGGGCTC	GTCTGCCAGTCAGCGGAGTGC
RTT18	GAGAGAGCCCAGAATGTCACT	GACATTCTGGGCTC	CTTTCGTAGAAG**a**CGAGT GACATTCTGGGCTC	GTCTGCCAGTCAGCGGAGTGC
RTT19	GAGAGAGCCCAGAATGTCACT	GACATTCTGGGCTC	GCTTTC**A**TAGAAG**a**CGAGT GACATTCTGGGCTC	GTCTGCCAGTCAGCGGAGTGC
RTT21	GAGAGAGCCCAGAATGTCACT	GACATTCTGGGCTC	CTGCTTTCGTAGAAG**a**CG AGTGACATTCTGGGCTC	GTCTGCCAGTCAGCGGAGTGC
RTT22	GAGAGAGCCCAGAATGTCACT	GACATTCTGGGCTC	CCTGCTTTC**A**TAGAAG**a**C GAGTGACATTCTGGGCTC	GTCTGCCAGTCAGCGGAGTGC
RTT24	GAGAGAGCCCAGAATGTCACT	GACATTCTGGGCTC	AGCCTGCTTTCGTAGAAG**a** CGAGTGACATTCTGGGCTC	GTCTGCCAGTCAGCGGAGTGC
RTT25	GAGAGAGCCCAGAATGTCACT	GACATTCTGGGCTC	CAGCCTGCTTTC**A**TAGAA G**a**CGAGTGACATTCT GGGCTC	GTCTGCCAGTCAGCGGAGTGC
RTT28	GAGAGAGCCCAGAATGTCACT	GACATTCTGGGCTC	CCTCAGCCTGCTTTC**A**TA GAAG**a**CGAGTGACATTC TGGGCTC	GTCTGCCAGTCAGCGGAGTGC
RTT31	GAGAGAGCCCAGAATGTCACT	GACATTCTGGGCTC	CCTCCTCAGCCTGCTTTC **A**TAGAAG**a**CGAGTGACA TTCTGGGCTC	GTCTGCCAGTCAGCGGAGTGC
RTT35	GAGAGAGCCCAGAATGTCACT	GACATTCTGGGCTC	TTGACCTCCTCAGCCTG CTTTC**A**TAGAAG**a**CGAG TGACATTCTGGGCTC	GTCTGCCAGTCAGCGGAGTGC
RTT39	GAGAGAGCCCAGAATGTCACT	GACATTCTGGGCTC	AGTATTGACCTCCTCAG CCTGCTTTC**A**TAGAAG**a**C GAGTGACATTCTGGGCTC	GTCTGCCAGTCAGCGGAGTGC
RTT42	GAGAGAGCCCAGAATGTCACT	GACATTCTGGGCTC	ACTCAGTATTGACCTCCTC AGCCTGCTTTCATAGAAG aCGAGTGACATTCTGGGCTC	GTCTGCCAGTCAGCGGAGTGC

**RTT length, PAM, and additional nucleotide modification experiments in HEK293T (1I)**				

+1A>G	GAGAGAGCCCAGAATGTCACT	GACATTCTGGGCTC	GCTTTC**A**TAGAAG**a**CGAG**C**	GTCTGCCAGTCAGCGGAGTGC
+2C>T	GAGAGAGCCCAGAATGTCACT	GACATTCTGGGCTC	GCTTTC**A**TAGAAG**a**CGA**A**T	GTCTGCCAGTCAGCGGAGTGC
+3C>T	GAGAGAGCCCAGAATGTCACT	GACATTCTGGGCTC	GCTTTC**A**TAGAAG**a**CG**g**GT	GTCTGCCAGTCAGCGGAGTGC
+4C>T	GAGAGAGCCCAGAATGTCACT	GACATTCTGGGCTC	GCTTTC**A**TAGAAG**a**C**A**AGT	GTCTGCCAGTCAGCGGAGTGC
+5G>A	GAGAGAGCCCAGAATGTCACT	GACATTCTGGGCTC	GCTTTC**A**TAGAAG**aT**GAGT	GTCTGCCAGTCAGCGGAGTGC
+7C>T	GAGAGAGCCCAGAATGTCACT	GACATTCTGGGCTC	GCTTTC**A**TAGAA**Aa**CGAGT	GTCTGCCAGTCAGCGGAGTGC
+8T>C	GAGAGAGCCCAGAATGTCACT	GACATTCTGGGCTC	GCTTTC**A**TAGA**G**G**a**CGAGT	GTCTGCCAGTCAGCGGAGTGC
+9T>C	GAGAGAGCCCAGAATGTCACT	GACATTCTGGGCTC	GCTTTC**A**TAG**g**AG**a**CGAGT	GTCTGCCAGTCAGCGGAGTGC
+10C>T	GAGAGAGCCCAGAATGTCACT	GACATTCTGGGCTC	GCTTTC**A**TA**A**AAG**a**CGAGT	GTCTGCCAGTCAGCGGAGTGC
+11T>C	GAGAGAGCCCAGAATGTCACT	GACATTCTGGGCTC	GCTTTC**A**T**G**GAAG**a**CGAGT	GTCTGCCAGTCAGCGGAGTGC
+12A>G	GAGAGAGCCCAGAATGTCACT	GACATTCTGGGCTC	GCTTTC**Ac**AGAAG**a**CGAGT	GTCTGCCAGTCAGCGGAGTGC
+14G>A	GAGAGAGCCCAGAATGTCACT	GACATTCTGGGCTC	GCTTT**TA**TAGAAG**a**CGAGT	GTCTGCCAGTCAGCGGAGTGC
+15A>G	GAGAGAGCCCAGAATGTCACT	GACATTCTGGGCTC	GCTT**c**C**A**TAGAAG**a**CGAGT	GTCTGCCAGTCAGCGGAGTGC
+16A>G	GAGAGAGCCCAGAATGTCACT	GACATTCTGGGCTC	GCT**C**TC**A**TAGAAG**a**CGAGT	GTCTGCCAGTCAGCGGAGTGC
+17A>G	GAGAGAGCCCAGAATGTCACT	GACATTCTGGGCTC	GC**C**TTC**A**TAGAAG**a**CGAGT	GTCTGCCAGTCAGCGGAGTGC
+18G>A	GAGAGAGCCCAGAATGTCACT	GACATTCTGGGCTC	G**T**TTTC**A**TAGAAG**a**CGAGT	GTCTGCCAGTCAGCGGAGTGC
+19C>T	GAGAGAGCCCAGAATGTCACT	GACATTCTGGGCTC	**A**CTTTC**A**TAGAAG**a**CGAGT	GTCTGCCAGTCAGCGGAGTGC
+3T>C	GAGAGAGCCCAGAATGTCACT	GACATTCTGGGCTC	CCTCCTCAGCCTGCTTTC **A**TAGAAG**a**CG**g**GT	GTCTGCCAGTCAGCGGAGTGC
+9T>C	GAGAGAGCCCAGAATGTCACT	GACATTCTGGGCTC	CCTCCTCAGCCTGCTTTC **A**TAG**g**AG**a**CGgGT	GTCTGCCAGTCAGCGGAGTGC
+12A>G	GAGAGAGCCCAGAATGTCACT	GACATTCTGGGCTC	CCTCCTCAGCCTGCTTT C**Ac**AGAAG**a**CGgGT	GTCTGCCAGTCAGCGGAGTGC
+15A>G	GAGAGAGCCCAGAATGTCACT	GACATTCTGGGCTC	CCTCCTCAGCCTGCTT**c**C **A**TAGAAG**a**CGgGT	GTCTGCCAGTCAGCGGAGTGC

**Five simultaneous mutations in HEK293T experiments (1J)**				

ADD MUT	GAGAGAGCCCAGAATGTCACT	GACATTCTGGGCTC	GC**A**TTC**A**TA**A**AAG**a** CGA**A**T	GTCTGCCAGTCAGCGGAGTGC

**Type of nucleotides at the target and in additional mutation in HEK293T experiments (1K)**				

+13C>T	GAGAGAGCCCAGAATGTCACT	GACATTCTGGGCTC	TTC**A**TAGAAG**a**CGAGT	GTCTGCCAGTCAGCGGAGTGC
+13C>G	GAGAGAGCCCAGAATGTCACT	GACATTCTGGGCTC	TTC**C**TAGAAG**a**CGAGT	GTCTGCCAGTCAGCGGAGTGC
+13C>A	GAGAGAGCCCAGAATGTCACT	GACATTCTGGGCTC	TTC**T**TAGAAG**a**CGAGT	GTCTGCCAGTCAGCGGAGTGC
+13C>T	GAGAGAGCCCAGAATGTCACT	GACATTCTGGGCTC	CCTCCTCAGCCTGCTT TC**A**TAGAAG**a**CGAGT	GTCTGCCAGTCAGCGGAGTGC
+13C>G	GAGAGAGCCCAGAATGTCACT	GACATTCTGGGCTC	CCTCCTCAGCCTGCTT TC**C**TAGAAG**a**CGAGT	GTCTGCCAGTCAGCGGAGTGC
+13C>A	GAGAGAGCCCAGAATGTCACT	GACATTCTGGGCTC	CCTCCTCAGCCTGCTT TC**T**TAGAAG**a**CGAGT	GTCTGCCAGTCAGCGGAGTGC
T +3T>C	GAGAGAGCCCAGAATGTCACT	GACATTCTGGGCTC	GCTTTC**A**TAGAAG**a**CG**g**GT	GTCTGCCAGTCAGCGGAGTGC
T +3T>G	GAGAGAGCCCAGAATGTCACT	GACATTCTGGGCTC	GCTTTC**A**TAGAAG**a**CG**c**GT	GTCTGCCAGTCAGCGGAGTGC
T +3T>A	GAGAGAGCCCAGAATGTCACT	GACATTCTGGGCTC	GCTTTC**A**TAGAAG**a**CG**t**GT	GTCTGCCAGTCAGCGGAGTGC
G +3T>C	GAGAGAGCCCAGAATGTCACT	GACATTCTGGGCTC	GCTTTC**C**TAGAAG**a**CG**g**GT	GTCTGCCAGTCAGCGGAGTGC
G +3T>G	GAGAGAGCCCAGAATGTCACT	GACATTCTGGGCTC	GCTTTC**C**TAGAAG**a**CG**c**GT	GTCTGCCAGTCAGCGGAGTGC
G +3T>A	GAGAGAGCCCAGAATGTCACT	GACATTCTGGGCTC	GCTTTC**C**TAGAAG**a**CG**t**GT	GTCTGCCAGTCAGCGGAGTGC
A +3T>C	GAGAGAGCCCAGAATGTCACT	GACATTCTGGGCTC	GCTTTC**T**TAGAAG**a**CG**g**GT	GTCTGCCAGTCAGCGGAGTGC
A +3T>G	GAGAGAGCCCAGAATGTCACT	GACATTCTGGGCTC	GCTTTC**T**TAGAAG**a**CG**c**GT	GTCTGCCAGTCAGCGGAGTGC
A +3T>A	GAGAGAGCCCAGAATGTCACT	GACATTCTGGGCTC	GCTTTC**T**TAGAAG**a**CG**t**GT	GTCTGCCAGTCAGCGGAGTGC
T +3T>C	GAGAGAGCCCAGAATGTCACT	GACATTCTGGGCTC	CCTCCTCAGCCTGCTTT C**A**TAGAAG**a**CG**g**GT	GTCTGCCAGTCAGCGGAGTGC
T +3T>G	GAGAGAGCCCAGAATGTCACT	GACATTCTGGGCTC	CCTCCTCAGCCTGCTTT C**A**TAGAAG**a**CG**c**GT	GTCTGCCAGTCAGCGGAGTGC
T +3T>A	GAGAGAGCCCAGAATGTCACT	GACATTCTGGGCTC	CCTCCTCAGCCTGCTTT C**A**TAGAAG**a**CG**t**GT	GTCTGCCAGTCAGCGGAGTGC
G +3T>C	GAGAGAGCCCAGAATGTCACT	GACATTCTGGGCTC	CCTCCTCAGCCTGCTTT C**c**TAGAAG**a**CG**g**GT	GTCTGCCAGTCAGCGGAGTGC
G +3T>G	GAGAGAGCCCAGAATGTCACT	GACATTCTGGGCTC	CCTCCTCAGCCTGCTTT C**c**TAGAAG**a**CG**c**GT	GTCTGCCAGTCAGCGGAGTGC
G +3T>A	GAGAGAGCCCAGAATGTCACT	GACATTCTGGGCTC	CCTCCTCAGCCTGCTTT C**c**TAGAAG**a**CG**t**GT	GTCTGCCAGTCAGCGGAGTGC
A +3T>C	GAGAGAGCCCAGAATGTCACT	GACATTCTGGGCTC	CCTCCTCAGCCTGCTTT C**t**TAGAAG**a**CG**g**GT	GTCTGCCAGTCAGCGGAGTGC
A +3T>G	GAGAGAGCCCAGAATGTCACT	GACATTCTGGGCTC	CCTCCTCAGCCTGCTTT C**t**TAGAAG**a**CG**c**GT	GTCTGCCAGTCAGCGGAGTGC
A +3T>A	GAGAGAGCCCAGAATGTCACT	GACATTCTGGGCTC	CCTCCTCAGCCTGCTTT C**t**TAGAAG**a**CG**t**GT	GTCTGCCAGTCAGCGGAGTGC

**Myoblast correction (1L)**				

+3T>C	GAGAGAGCCCAGAATGTCACT	GACATTCTGGGCTC	GCTTTC**G**TAGAAG**a**CG**g**GT	GTCTGCCAGTCAGCGGAGTGC
+9T>C	GAGAGAGCCCAGAATGTCACT	GACATTCTGGGCTC	GCTTTC**G**TAG**g**AG**a**CGAGT	GTCTGCCAGTCAGCGGAGTGC
+12A>G	GAGAGAGCCCAGAATGTCACT	GACATTCTGGGCTC	GCTTTC**Gc**AGAAG**a**CGAGT	GTCTGCCAGTCAGCGGAGTGC
+15A>G	GAGAGAGCCCAGAATGTCACT	GACATTCTGGGCTC	GCTT**c**C**G**TAGAAG**a**CGAGT	GTCTGCCAGTCAGCGGAGTGC
+3T>C	GAGAGAGCCCAGAATGTCACT	GACATTCTGGGCTC	CCTCCTCAGCCTGCTTTC **G**TAGAAG**a**CG**g**GT	GTCTGCCAGTCAGCGGAGTGC
+9 T>C	GAGAGAGCCCAGAATGTCACT	GACATTCTGGGCTC	CCTCCTCAGCCTGCTTTC **G**TAG**g**AG**a**CGgGT	GTCTGCCAGTCAGCGGAGTGC
+12A>G	GAGAGAGCCCAGAATGTCACT	GACATTCTGGGCTC	CCTCCTCAGCCTGCTTT C**Gc**AGAAG**a**CGgGT	GTCTGCCAGTCAGCGGAGTGC
+15A>G	GAGAGAGCCCAGAATGTCACT	GACATTCTGGGCTC	CCTCCTCAGCCTGCTT **c**C**G**TAGAAG**a**CGAGT	GTCTGCCAGTCAGCGGAGTGC

Nucleotides in boldface represent the modifications to induce using the primer binding site (PBS) and reverse transcriptase template (RTT).

**Table 2:** Primer sequences for PCR and sequencing

Primer names	Sequences
Pr PCR Fwd	GCACTCTTATCTCAATGAGAGG
Pr PCR Rev	AGGTGATCTTGGAGAGAGTC
Pr Sanger sequencing	ATCACCTCAGCTTGGCGCAGCT
Pr deep sequencing Fwd	**ACACTGACGACATGGTTCTACA** CGTTAATCAGTAGGTTACCCTC
Pr deep sequencing Rev	**TACGGTAGCAGAGACTTGGTCT** TTGAGGTCCAGCTCATCCGT

Sequences in boldface represent specific barcode sequences for deep sequencing.

### The use of SpCas9 variants to induce the same target nucleotide modification

Since the +13 position was less favorable for prime editing because it is far from the nick site, we decided to test other SpCas9n variants, which used a PAM closer to the target nucleotide than when using the normal NGG SpCas9 PAM. We thus constructed two plasmid variants, the SpCas9n-VQR recognizing an NGAN PAM^28^ and the SpCas9n-RY recognizing the NNN PAM sequence (N being any nucleotide)^29^ to make, respectively, the PE2-VQR and the PE2-SpRY.^30^ The PE2-VQR was making it possible to induce the same nucleotide modification at +1 instead of at +13 with NGG PAM, and the PE2-RY was permitting us to make the modification at +3 considering the combination of nucleotide in PAM sequence that showed better results in other studies.^29^^,^^30^ We designed three other pegRNAs for each of these two variants, namely pegRNAa, pegRNAb, and pegRNAc, with different reverse transcription template (RTT), primer binding site (PBS), and spacer sequences (Table 1, rows 1B). The codified pegRNAa, pegRNAb, and pegRNAc for PE2-NGG variant correspond to the same sequences described in Table 1 (rows 1A). The PE2-NGG, PE2-VQR, and PE2-SpRY variants were each co-transfected with one of the appropriate pegRNA plasmids in HEK293T cells. Three days after transfection the cells were harvested, and a partial DNA sequence of exon 59 was PCR amplified and Sanger sequenced. The results showed editing percentages of up to 6.5% ± 0.7%, 5.5% ± 0.5%, and 5.5% ± 0.7%, respectively with PE2-NGG, PE2-VQR, and PE2-RY (Figure 1B). Thus, PE2-NGG remained the best editing method although the target nucleotide was at +13 from the nick site.

### Modification of the PAM to increase the edit of the target nucleotide

Since the editing percentages were only around 6%, we hypothesized that changing the second G nucleotide of the SpCas9n CGG PAM, which is an arginine codon, into a T nucleotide to form the CGT codon, which remains an arginine codon, could improve the editing efficiency by preventing the DNA to be nicked again by SpCas9n after a previous successful modification. We designed three new pegRNAs (pegRNA1′, pegRNA2′, and pegRNA3′) (Table 1, rows 1C) containing both the intended nucleotide modification at +13 and an additional PAM modification at +6. The results showed editing percentages of 7.3% ± 0.5%, 6.5% ± 0.7%, and 5.5% ± 0.7% for the PE2 strategy, which represented 1.2-fold increase for pegRNA1′ and pegRNA2′ compared with the pegRNAs not mutating the PAM (Figure 1C). We also used an additional sgRNA to nick at +62 for the PE3 strategy leading to 11% ± 1%, 6.5% ± 0.7%, and 4.5% ± 0.7% editing percentage, which represented a 1.4-fold increase only for the pegRNA1′ (Figure 1C). The results also highlighted a high editing percentage of 36% ± 4.2% for the G nucleotide of the CGG PAM to be changed into T nucleotide (Figure 1C). This confirmed that the frequency of nucleotide editing is very high for nucleotides located near the nick site.

### Checking whether the position of the intended mutation is influenced by the modification induced in the PAM sequence

**Figure 2:**
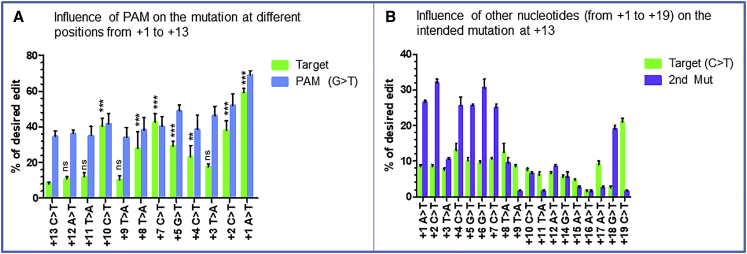
Influence of the PAM nucleotide or other nucleotides in the target (A) Results obtained when the guanine nucleotide at +6 from the PAM is modified into a thymine simultaneously with the modification of one nucleotide located at positions spanning +1 to +13 from the nick induced by the SpCas9n. Position +13 is indicated in the red square in Figure 1D. At this position 13, the CGA codon is to be changed to a TGA codon. (B) Results obtained when modifications are done from +1 to +19 while modifying simultaneously the target nucleotide at +13. These experiments were done in independent triplicates (n = 3). All the editing percentages at different targets were compared with the modification at +13. The p values were calculated using the non-parametric Mann-Whitney U test. ∗∗∗p < 0.001; ∗∗p = 0.01; ns, non-significant difference.

To verify whether the intended nucleotide modification efficiency is influenced by the PAM modification and the distance from the PAM or from the nick site, we decided to modify each nucleotide individually from +1 to +13 while maintaining the PAM edit at position +6 (Figure 2A). We used the pegRNA1′, which was the best among the three pegRNAs, and the sgRNA to induce a second nick at +62. We designed 12 other pegRNAs (Table 1, rows 1D) from the pegRNA1′ to induce at the position +1 the modification of A to T (+1A>T), +2C>T, +3T>A, +4C>T, +5G>T, +7C>T, +8T>A, +9T>A, +10C>T, +11T>A, +12A>T, and +13C>T but always maintaining +6G>T in the PAM sequence. The highest editing rate was observed with +1A>T that showed 59.3% ± 3.7% and 69% ± 4.3% modification, respectively for the intended modification (+1A>T) and the PAM modification (+6G>T) (Figure 2A). We observed that the efficiency varies depending on the type of nucleotide to be changed, the distance from the nicking site, and the modification of the PAM sequence. These results also confirmed that the editing efficiency decreased progressively for nucleotides to be changed from positions +11 to +13.

### Checking whether the modification of nucleotides other than the PAM could influence the nucleotide mutation at +13

From the same pegRNA1′, we designed 18 other pegRNAs (Table 1, rows 1E) to modify each nucleotide from position +1 to +19 while maintaining the intended edit at +13 to verify whether the modification of nucleotides other than the PAM sequence (+6G>T) could influence our intended modification at +13 using the PE3 strategy (Figure 2B). The results showed 21% ± 1.4% of desired modification at +13 when a G was changed to A simultaneously at position +19 (Figure 2B). This percentage was on average 2-fold higher than the results obtained by modifying simultaneously only the PAM sequence. These results confirmed that the editing efficiency at the target (+13C>T) is influenced by the modification of other nucleotides around it. Unfortunately, none of the second mutations introduced around the desired modification with the highest efficiency at +13 could mediate a silent mutation. Thus, our best option remained the PAM modification that can mediate a silent mutation of the arginine (R) codon.

### Verifying the influence of the type of nucleotide to change in the PAM sequence

**Figure 3:**
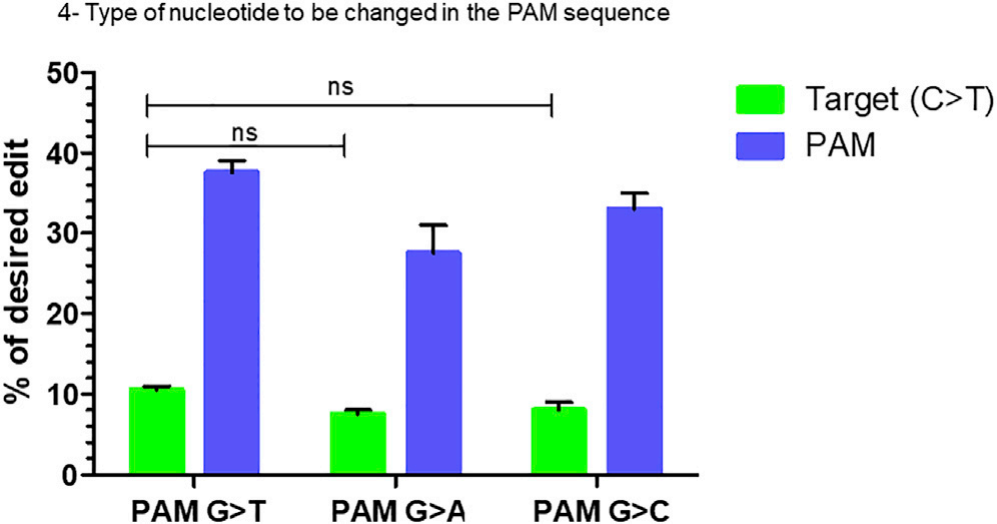
The type of nucleotide to be changed at the target This figure shows the difference in editing efficiency to induce c.8713C>T mutation in exon 59 of *DMD* gene while also changing one nucleotide of the PAM sequence. The PAM sequence CGG is changed respectively to CGT, CGA, and CGC, which are all coding for the arginine amino acid. Each of these modifications was done simultaneously with the modification of the target at +13 changing C to T. These experiments were done in independent triplicates (n = 3). The differences in editing efficiency at the target for the different modifications in PAM sequences were not statistically significant (ns).

To answer the question as to whether the type of nucleotide change in the PAM sequence can have more or less influence on the desired modification at +13, we designed different pegRNAs (Table 1, rows 1F) to check the combination of different nucleotides at position +6 in the PAM sequence while maintaining the desired +13C>T mutation. The different possible combinations were G>T, G>A, and G>C. The PE3 results showed average editing percentages of 10.5% ± 0.7%, 7.5% ± 0.7%, and 8% ± 1.4%, respectively for C>T modification at +13 and 37.5% ± 2.1%, 27.5% ± 4.1%, and 33% ± 2.8%, respectively for the G>T, G>A, and G>C modifications in the PAM sequence (Figure 3).

### Modification of RTT length to influence the edit at +13

**Figure 4:**
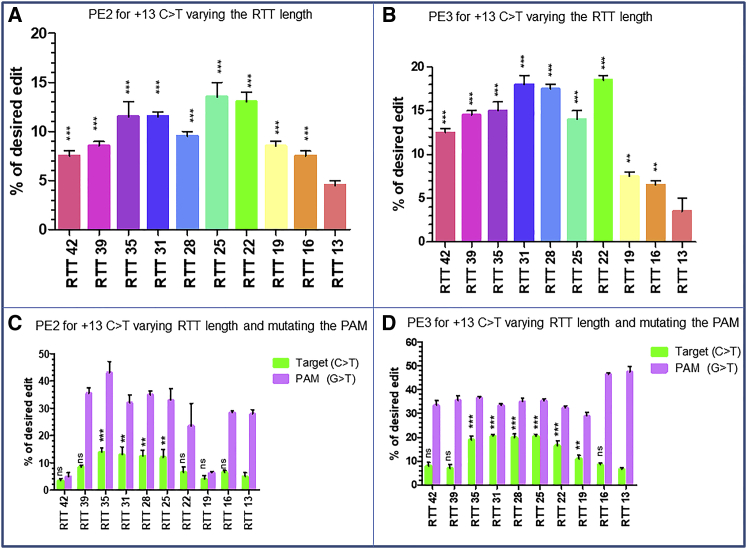
Influence of the RTT length on the target (A and B) The PE2 (A) and PE3 (B) results for the introduction of c.8713C>T mutation in exon 59 of *DMD* gene when the RTT length varies from 13 (RTT 13) to 42 (RTT 42). The difference was not statistically significant (ns) either for PE2 or PE3 using the Kruskal-Wallis test. (C and D) The PE2 (C) and PE3 (D) results when the RTT length varies from RTT 13 to RTT 42. The modification at the target (green) is done simultaneously with the modification of the PAM sequence (purple). The experiments were done in triplicates (n = 3). The p values were calculated using the Kruskal-Wallis test. The editing percentages were compared between RTT13 and other RTTs for the mutation at the target site. ∗∗∗p = 0.001; ∗∗p = 0.01; ∗p < 0.05; ns, non-significant difference.

To verify whether modification of the RTT length could increase the results of the modifications at +13, we designed ten pegRNAs from the initial pegRNA1 (RTT16, PBS14) with RTT length varying from 13 (RTT13) to 42 (RTT42) (Table 1, rows 1G). The PE2 strategy results showed an increased in editing efficiency up to 13.5% ± 2.1% for RTT25 (Figure 4A). The PE3 results with the same sgRNA at +62 showed up to 18.5% ± 0.7% modification (Figure 4B). We also designed ten other RTT sequences carrying both the modification in the PAM sequence at position +6 and at the target at +13 (Table 1, rows 1H). The PE2 results indicated up to 13% ± 2.8% modification at the target with the best RTT length ranging from RTT25 to RTT35 (Figure 4C). The PE3 results showed a pick of 20.5% ± 0.7% modification at the target nucleotide (+13C>T) for RTT31 (Figure 4D), which was 7% higher than the pick observed with the PE3 strategy without modification in the PAM (Figure 4C). From these observations, we reasoned that the RTT variation and an additional mutation could have an influence in the editing efficiency at the target.

### Additional mutations in the RTT sequence

**Figure 5:**
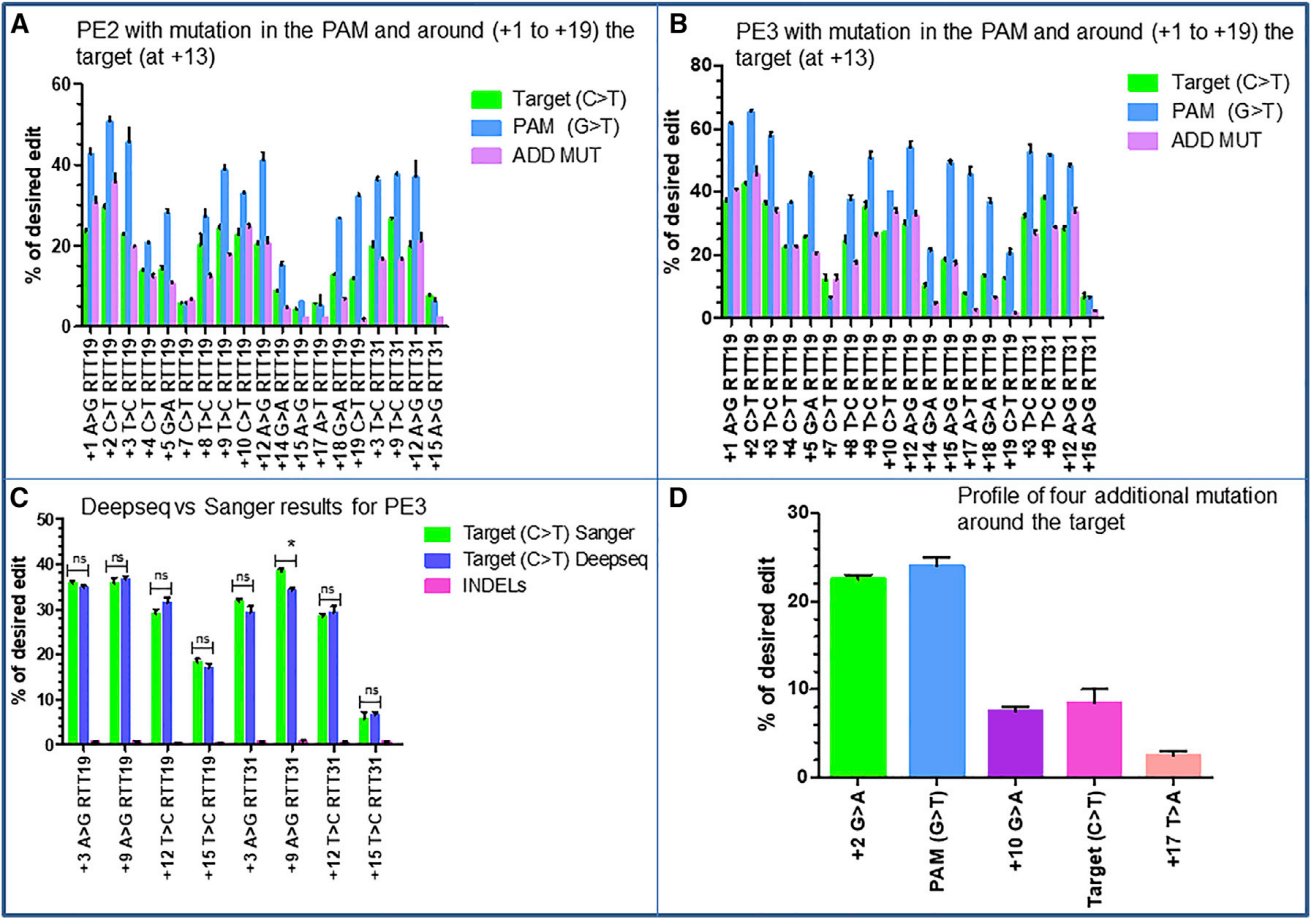
Influence of simultaneous additional mutations on the target (A and B) Variations in editing efficacy for PE2 (A) and PE3 (B) strategies for the introduction of c.8713C>T mutation in exon 59 of *DMD* gene when the PAM sequence and a third additional nucleotide (ADD MUT) are simultaneously modified. The three mutations are all introduced by a pegRNA. The third mutation is introduced at different positions (from +1 to +15) in RTT19 and RTT31. (C) Illumina deep sequencing and Sanger sequencing results for PE3 strategy (shown in B) when the modification at the target is done simultaneously with the modification in the PAM sequence and the introduction of the third additional silent mutations at different positions of RTT19 and RTT31. ∗p = 0.01 using the non-parametric Mann-Whitney U test. ns, non-significant difference. (D) Individual results for the combination of five different mutations at different positions of the RTT19 sequence. The experiments were done in independent triplicates (n = 3).

To check whether additional mutation in the RTT sequence could increase the target modification at +13, we designed 19 new pegRNA sequences from the initial pegRNA1 (Table 1, rows 1I). Each pegRNA contained three mutations always including the target mutation at +13, the mutation in the PAM, and with only the third additional mutation changing at different positions spanning from +1 to +19. Among the 19 designed pegRNAs, four had the RTT31 because it exhibited the highest editing percentage in previous results. For the four pegRNAs with RTT31, the third mutation was selected to induce a silent mutation respectively at positions +3 changing a T to C (+3T>C RTT31), +9 changing a T to C (+9T>C RTT31), +12 changing an A to G (+12A>G RTT31), and +15 changing an A to G (+15A>G RTT31). On the other hand, the 15 remaining pegRNAs contained RTT19 with the third mutation spanning from +1 to +19. The PE2 results with RTT19 showed up to 28% ± 0.7% editing at the target, indicating that the additional mutation in the RTT increased by 2.7-fold the desired modification at +13 (Figure 5A). The PE2 results with RTT31 also showed an increase of 1.6-fold with an editing percentage of up to 25% ± 0.7%. The PE3 results showed up to 42% ± 0.7% modification at the target for pegRNAs with RTT19 and RTT31 (Figure 5B). We observed that the target modification at +13 was significantly influenced by the simultaneous modification of the PAM sequence and an additional mutation at different positions around the target. The distance from the target and the type of nucleotide modification seemed to play a role in that efficiency.

From the PE3 strategy, we selected harvested samples transfected with pegRNAs in which the third additional mutation could induce a synonymous mutation. The results were analyzed by Illumina deep sequencing. These included +3T>C RTT19, +9T>C RTT19, +12A>G RTT19, +15A>G RTT19, +3T>C RTT31, +9T>C RTT31, +12A>G RTT31, and +15A>G RTT31. The Illumina sequencing results were almost identical to those obtained with Sanger sequencing (Figure 5C). Deep sequencing of these amplicons showed average indels of 0.9% with a maximum of 1.5% recorded with +15A>G RTT31.

### Combinations of more than two additional mutations

Since the combination of two additional mutations around the target significantly influenced its efficiency, we decided to check whether more than two mutations could give more interesting results. We designed a pegRNA (Table 1, row 1J) containing four mutations in addition to the target mutation at +13. A total of five simultaneous mutations were inserted in a single pegRNA (RTT19, PBS14) including: the target (+13C>T), the PAM (+6G>T), the third additional mutation (+2C>T), the fourth additional mutation (+10C>T), and the fifth additional mutation (+17A>T). The PE3 strategy results showed that the interactions between all these mutations decreased the editing efficiency at +13; moreover, each individual nucleotide mutation was also decreased (Figure 5D).

### Checking whether the type of nucleotide can influence the modification of the target

**Figure 6:**
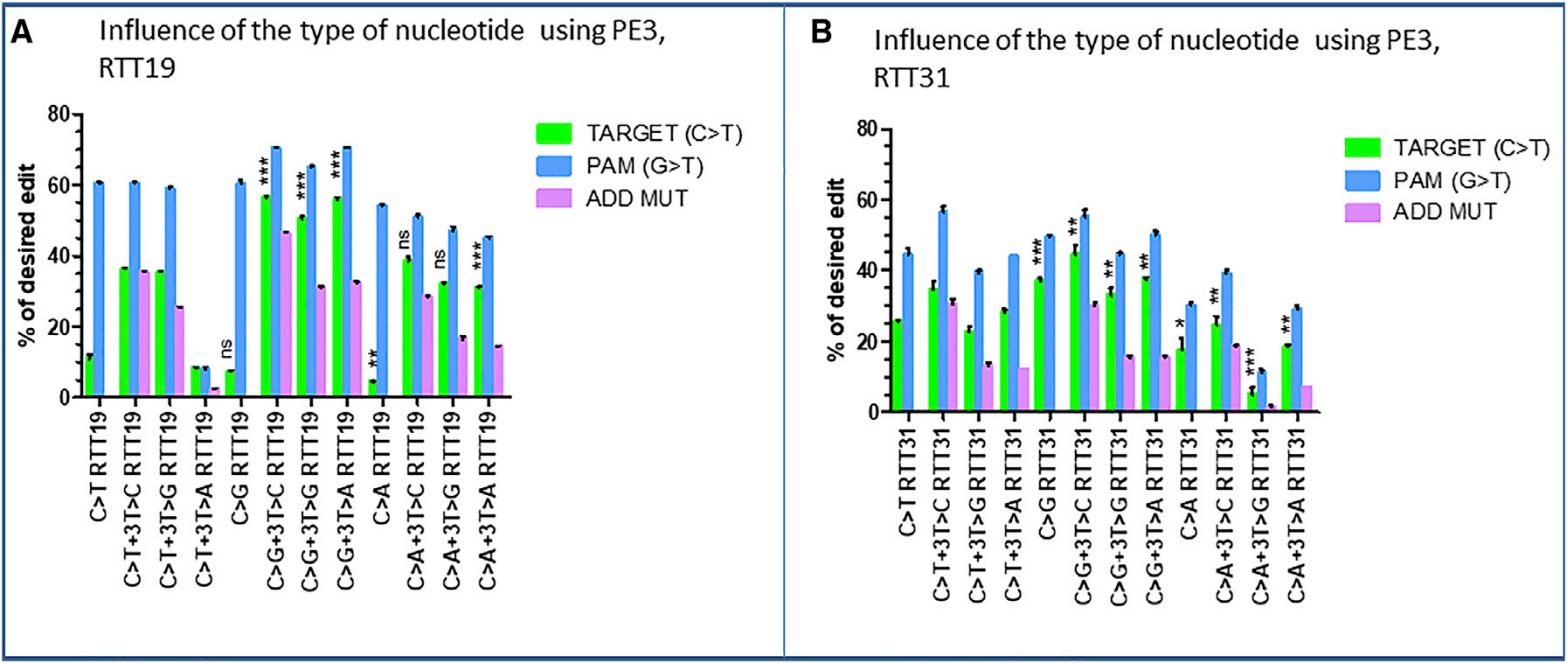
Influence of the type of nucleotide to be changed at the target This figure shows the PE3 results with different pegRNAs having RTT19 (A) and RTT31 (B). Here, the C nucleotide at the target site is changed either to T (C>T), G (C>G), or A (C>A). Each time these mutations are done, the PAM sequence at the position +6 and the third additional nucleotide (ADD MUT) at the position +3 are simultaneously changed as indicated in the x axis of the graph. The experiments were done in independent triplicates (n = 3). The p values were calculated using the non-parametric Mann-Whitney U test . The C>T groups were compared with C>G and C>A groups. ∗∗∗p = 0.0001, ∗∗p = 0.001, ∗p = 0.01, and p > 0.05 (ns) for 5% confidence interval.

We decided to change the C nucleotide at the +13 target to either an A, a G, or a T. We designed different pegRNAs with RTT19 to verify the potential effects on the nucleotide changes (Table 1, rows 1K). While changing the C to T at position +13, we also simultaneously changed at the position +3 either the T to C (C>T +3T>C RTT19), T to G (C>T +3T>G RTT19), or T to A (C>T +3T>A RTT19). Similarly, we also changed the C to G and the C to A at +13, while simultaneously changing at the position +3 either the T to C (C>G +3T>C RTT19 and C>A +3T>C RTT19), T to G (C>G +3T>G RTT19 and C>A+3T>G RTT19), or T to A (C>G +3T>A RTT19 and C>A +3T>A RTT19). With the PE3 results, we observed up to 36.5% ± 0.5%, 58% ± 1.1%, and 40% ± 1.8% editing efficiency, respectively for C to T, C to G, and C to A modification at the target (Figure 6A). This indicated that the type of nucleotide to be changed at the target and the type of nucleotides at the additional mutation sites highly influenced the editing efficiency. The same experiment was done with different pegRNAs with RTT31. The results were lower than those observed with pegRNAs with RTT19 Figure 6B).

### Correction of the C to T mutation at +13

**Figure 7:**
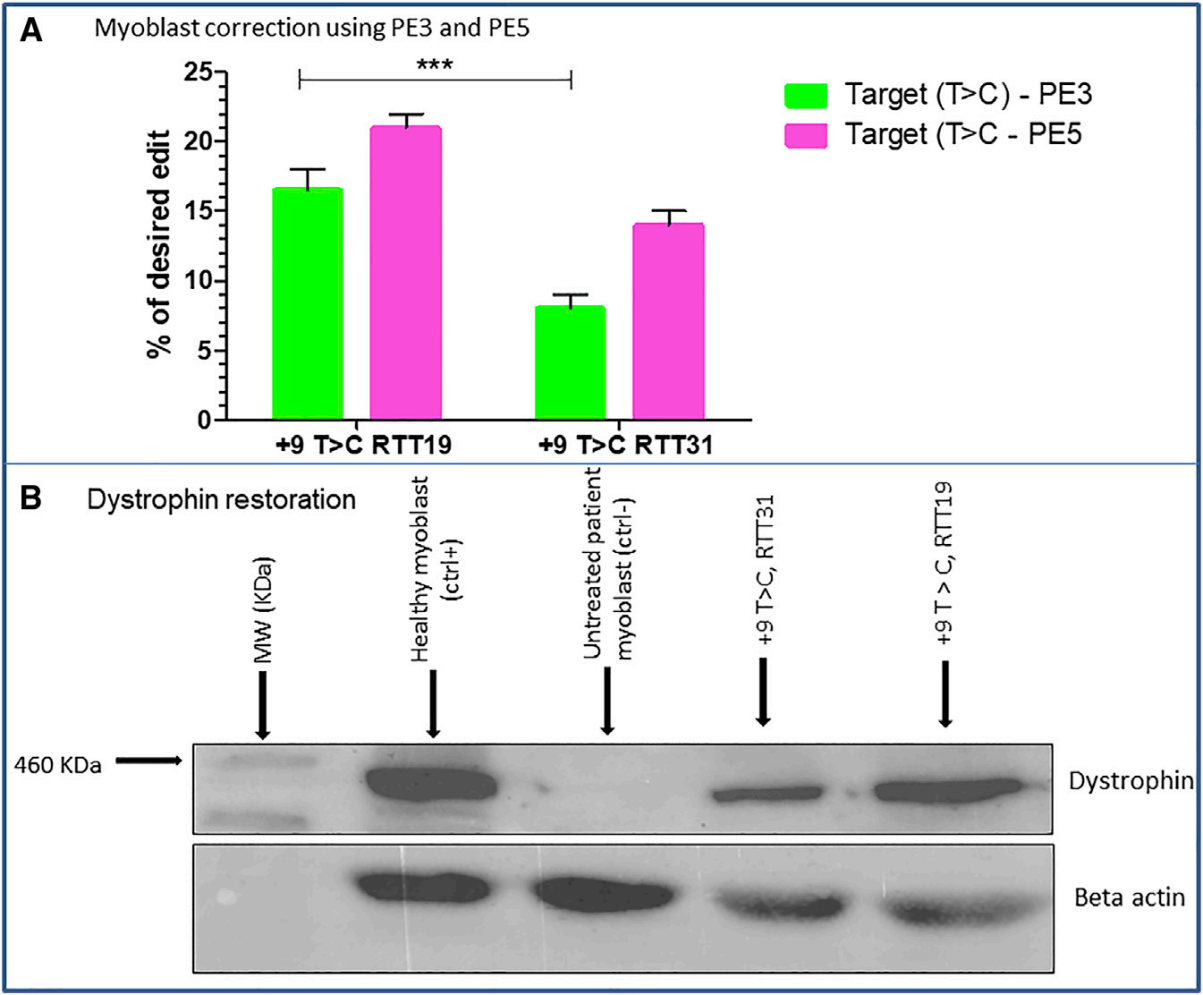
Correction of *DMD* c.8713C>T mutation (A) Editing percentage for PE3 and PE5 strategies in human myoblasts for the correction of c.8713C>T mutation in exon 59 of *DMD* gene. The RTT19 and RTT31 used here permitted us to induce the desired modification (T>C) at +13 while modifying simultaneously the PAM sequence (G>T) at +6 and the additional nucleotide (T>C) at +9. The experiments were done in triplicates (n = 3). The mean editing percentage at the target site was statistically significant with (∗∗∗p < 0.001) using the non-parametric Mann-Whitney U test. (B) Western blot resulting from 20 μg of total protein obtained by the lysis of myotubes from the culture plate. It indicates the molecular weight marker (460 kDa), the negative control sample (Ctrl−), which used myoblasts with point mutation in exon 59, the positive controls (Ctrl+), which were healthy human myoblasts, and the samples treated with the pegRNAs described in (A).

With the collaboration of the Institut de Myologie de Paris, we obtained a human myoblast cell line carrying the c.8713C>T point mutation. For the correction of that mutation, we selected two pegRNAs (Table 1, rows 1L) from the different optimizations we made to introduce the same mutation in HEK293T cells to create a stop codon. We chose the +9T>C RTT19 and +9T>C RTT31, which both gave about 35% modification in HEK293T using the PE3 strategy. These pegRNAs permitted, while inducing the modification at the target (+13C>T), introduction of a silent mutation in the PAM sequence (+6G>T) and additional mutation (+9T>C). In these pegRNAs, we changed only the nucleotide at the target position to correct the mutation instead of creating the mutation. In addition to the PE3 strategy, which uses the pCMV-PE2 plasmid with the pegRNA and sgRNA plasmids, we also tested the PE5 strategy recently described by Chen et al.,^24^ which uses the pCMV-PEmax-P2A-hMLH1dn with the pegRNA and sgRNA plasmids. After proliferation, 100,000 human myoblasts carrying the c.8713C>T mutation were electroporated separately with 1 μg of each of the two selected and modified pegRNAs and 1 μg of pCMV-PE2 plasmid for PE3 strategy or pCMV-PEmax-P2A-hMLH1dn for PE5 strategy. Three to five days after electroporation, cells were collected and separated into two parts. One part was immediately used for DNA extraction, PCR amplification, and sequencing through the Sanger method. The results for the PE3 strategy showed 17% ± 2.1% and 8% ± 1.4% editing, respectively, for the +9T>C RTT19 and +9T>C RTT31 pegRNAs (Figure 7A). The PE5 strategy showed 21% ± 1.4% and 14% ± 1.4% editing efficiency, respectively for the +9T>C RTT19 and +9T>C RTT31 pegRNAs (Figure 7A). These represented 1.2-fold and 1.7-fold increases, respectively with +9T>C RTT19 and +9T>C RTT31 pegRNAs using the PE5 strategy. The other part of the harvested cells for the PE5 strategy was used for myotube formation through the fusion of myoblasts to verify whether expression of the dystrophin protein was restored. Western blotting carried out with 20 μg of total protein showed dystrophin expression of 42% and 31%, respectively for the two pegRNAs (Figure 7B).

## Discussion

The prime editing technique for genome editing is constantly being optimized.^23^^,^^24^^,^^31^ Our results show that increasing the RTT length for a target mutation site that is far from the nick site improved the editing efficiency. It has been demonstrated that some target sites prefer long RTT sequences while others prefer short RTT sequences.^22^ However, in combination with the RTT length, our results showed that the addition of one or many nucleotide modifications at different sites from the intended edit significantly improved the editing outcome. Chen et al.^24^ also indicated that inserting 1–4 silent mutations between the positions +7 and +14 improved by 1.5-fold the edit at the position +6.

We demonstrated that the position of the target mutation, the type of nucleotide to be modified at that position, and the type and position of the additional nucleotide mutation around the desired target highly influenced the nucleotide change efficiency at the target. The mechanism by which this interaction occurs is currently poorly understood. The additional mutations around the target may play a role during the mismatch repair mechanism favoring the installation of the intended modification.^24^ These additional mutations can interact by increasing or decreasing the editing efficiency of one or the other nucleotide. A negative impact on the mutation at +13 and on each additional nucleotide taken individually was observed when four additional mutations that previously individually showed high editing percentages were inserted simultaneously by the same RTT sequence. Since the mismatch repair varies by mismatch type,^32^ the type and the position of the additional mutation could repress or favor its installation. The distance between the nucleotide to be modified and additional mutations also plays a role during the process.

This optimized strategy by modifying the RTT sequence increased by 3.8-fold the intended modification at position +13. When the nucleotide at the target in the non-PAM strand was changed to C instead of A in our case, we observed a 6.6-fold increase using a pegRNA with only one nucleotide difference (C>G instead of C>T). This indicates that point mutation can be corrected in the *DMD* gene more effectively when taking into consideration the type of nucleotide to be changed and the possibility of inserting one or more additional silent mutations.

Combining this strategy with the PEmax-hMLH1dn strategy,^24^ we obtained up to a 1.7-fold increase in editing efficiency for the correction of c.8713C>T mutation. The PEmax-hMLH1dn strategy permits the disruption of *hMLH1* mismatch repair gene, which acts at the genomic damage checkpoint to stabilize the MutS-DNA complex^33^ and favor the installation of the intended mutation. The improvement induced by modifications of repair factors, among which MLH1 gene is the best candidate, might vary depending on cell lines and the type of edit.^34^ This strategy permitted us to achieve 22% modification in human myoblasts for the correction of c.8713C>T mutation. This percentage represents a good modification level for the *DMD* gene. In fact, the dystrophin nuclear domain contains about 30 nuclei and is about 439 μm long.^35^, ^36^, ^37^ Considering that a muscle fiber is made of thousands of nuclei, the correction in one nucleus in a nuclear domain (approximately 3%) could be enough for a phenotypic improvement.

## Materials and methods

### Plasmids

The pCMV-PE2, the pCMV-PEmax-P2A-hMLH1dn and pU6-pegRNA-GG-acceptor plasmids were a gift from David Liu (Addgene plasmids #132775, #174828, and #132777). Cloning in these plasmids was done as described by Anzalone et al.^22^ Oligonucleotides used for the construction of pegRNAs were purchased from IDT (Coralville, IA, USA).

### Cell culture

HEK293T were grown in DMEM-HG medium (Wisent, Saint-Jean-Baptiste, QC, Canada) supplemented with 10% fetal bovine serum (FBS) (Wisent) and 1% penicillin-streptomycin (Wisent) at 37°C with 5% CO_2_ in a humidified incubator. The day before transfection, cells were detached from the flask with a trypsin-EDTA solution (Sigma-Aldrich Canada, Oakville, ON, Canada) and counted. Detached cells were plated on a 24-well plate at a density of 60,000 cells per well with 1 mL of culture medium. On the transfection day, the medium was replaced with 500 μL of fresh medium. Cells were transfected with 1 μg of total DNA (500 ng of each plasmid when co-transfection was required) with Lipofectamine 2000 (Invitrogen, Carlsbad, CA, USA) according to the manufacturer’s instructions. The medium was changed to 1 mL of fresh medium 24 h later, and cells were maintained in incubation for 72 h before genomic DNA extraction.

The human myoblasts were grown in a home-made medium made of 4 volumes of DMEM-HG medium for 1 volume of medium 199 (Invitrogen) supplemented with 25 μg/mL fetuin (Life Technologies, Carlsbad, CA, USA), 5 ng/mL human epidermal growth factor (Life Technologies), 0.5 ng/mL basic fibroblast growth factor (Life Technologies), 5 μg/mL insulin (Sigma-Aldrich Canada, 91077C-1G), 0.2 μg/mL dexamethasone (Sigma-Aldrich Canada). A total of 2 μg of plasmids (1 μg of pCMV-PE2 or pCMV-PEmax-P2A-hMLH1dn plasmid and 1 μg of pU6-GG-acceptor plasmid containing the pegRNA sequence and the sgRNA for PE3) were added to 100,000 human myoblasts and electroporated with the Neon Transfection System following the program 1,100 V/20 ms/2 pulses. These electroporated cells were placed in one well of a 24-well culture plate containing 500 μL of the home-made medium. The electroporation medium was changed to 1 mL of fresh medium after 24 h, and cells were detached with trypsin and harvested in 1 mL of culture medium for the next 48 h. Half of the harvested cells was used for DNA extraction, and the remaining volume was transferred to one well of a 6-well culture plate containing 2 mL of the home-made medium. At 80%–90% confluency, the medium was changed to 2 mL of DMEM containing 1% FBS to induce myoblast fusion to form myotubes, which were harvested a few days later for western blot analysis of dystrophin.

### Genomic DNA preparation, amplification, and sequencing

HEK293T cells were detached from wells directly with up-and-down pipetting of the culture medium and transferred in 1.5-mL Eppendorf tubes. Human myoblasts were detached using trypsin-EDTA solution (Sigma-Aldrich Canada) and collected in 1 mL of the original medium. HEK293T cells or human myoblasts were spun for 5 min at 9,000 rpm in a microcentrifuge at room temperature. Cell pellets were washed once with 1 mL of 1× phosphate-buffered saline and spun again for 5 min at 9,000 rpm. Genomic DNA was prepared using the DirectPCR Lysis Reagent (Viagen Biotech, Los Angeles, CA, USA). In brief, 50 μL of DirectPCR Lysis Reagent containing 0.5 μL of a proteinase K solution (20 mg/mL) was added to each cell pellet and incubated overnight at 56°C followed by another incubation at 85°C for 45 min and centrifugation at 13,000 rpm for 5 min; 1 μL of each genomic DNA preparation (supernatant) was used for the PCR reaction. For each primer set (Table 2), PCR temperature cycling was as follows: 98°C for 30 s and 35 cycles of 98°C for 10 s, 60°C for 20 s, and 72°C for 45 s. A final extension at 72°C for 5 min was also performed. We used Phusion High-Fidelity DNA polymerase from Thermo Fisher Scientific (Waltham, MA, USA) for all PCR reactions. Five microliters of amplicons was electrophoresed in 1× Tris/borate/EDTA buffer on 1% agarose gel to control the PCR reaction qualities and to make sure that only one specific band was present.

### Sanger sequencing

Amplicons from PCR (i.e., the remaining 45 μL) were sent to the sequencing platform of the CHU de Québec Research Center for Sanger sequencing. An internal primer (Table 2) was used for polymerization using the BigDye Terminator v3.1 (Thermo Fisher Scientific). Sequences were analyzed with the EditR online program (http://baseeditr.com)^38^ to determine the editing percentage in the targeted region of the *DMD* gene.

### Deep sequencing analysis

Deep sequencing samples were prepared by a PCR reaction (as described above) with special primers containing a barcode sequence to permit the subsequent deep sequencing (Table 2). PCR samples were sent to the Genome Québec Innovation Center at McGill University to sequence amplicons with the Illumina sequencer. Roughly 6,000–10,000 reads were obtained per sample. Illumina sequencing results were analyzed with the CRISPResso2 online program (https://crispresso.pinellolab.partners.org/).^39^

### Western blot analysis

Myotubes were detached directly from a culture plate with 400 μL of lysis buffer supplemented with protease inhibitors. One microliter of extracted proteins and different concentrations of BSA (used as standard) were put onto a nitrocellulose membrane and colored with amino black 10B. The membrane was scanned by the ChemiDoc XRS+ system (Bio-Rad Laboratories, Hercules, CA, USA) and quantified using ImageLab 6.0.1 software (Bio-Rad) according to the manufacturer’s instructions. Twenty micrograms of extracted protein samples was separated by SDS-PAGE (4%–7%) and transferred onto a polyvinylidene fluoride membrane. A mouse monoclonal antibody against dystrophin (clone MANDYS8; Abnova, Taipei, Taiwan) and the mouse β-actin antibody against β-actin (Thermo Fisher Scientific) were used for immunoblotting analysis. Horseradish peroxidase-conjugated goat anti-mouse (Thermo Fisher Scientific) was used as secondary antibody. The membrane was developed using Clarity Western ECL substrate (Bio-Rad) and scanned by the ChemiDoc XRS+ system (Bio-Rad).

### Statistical analysis

Data were analyzed using the GraphPad PRISM 5.0 software package (Graph Pad Software, La Jolla, CA, USA). Comparisons between the mean editing percentage among different groups were performed using the Mann-Whitney non-parametric U test. Comparisons between single pegRNAs were performed using Kruskal-Wallis one way ANOVA. A p value of <0.05 was considered statistically significant for a 5% confidence interval.

## Data Availability

The datasets generated during and/or analyzed during the current study are available from the corresponding author on reasonable request.
